# C26 and CT26 colorectal cancer models exhibit divergent cachexia phenotypes, intramuscular inflammation, and protein turnover signaling

**DOI:** 10.64898/2026.04.21.719997

**Published:** 2026-04-24

**Authors:** Xinyue Lu, Hanan Tlais, Hamood Rehman, Ashtyn N. Martens, Abigail L. Hartz, Vandré C. Figueiredo, James F. Markworth

**Affiliations:** 1 Department of Animal Sciences, College of Agriculture, Purdue University, West Lafayette, IN, USA.; 2 Interdepartmental Nutrition Program (INP), College of Health and Human Sciences, Purdue University, West Lafayette, IN, USA.; 3 Center for Aging and the Life Course (CALC), Purdue University, West Lafayette, IN, USA.; 4 Department of Biological Sciences, Oakland University, Rochester, MI, USA.; 5 Purdue Institute of Inflammation, Immunology, and Infectious Disease (PI4D), Purdue University, West Lafayette, IN, USA.; 6 Indiana Center for Musculoskeletal Health (ICMH), Indiana University, Indianapolis, IN, USA.

## Abstract

Colorectal cancer (CRC) cachexia induces skeletal muscle dysfunction, impeding quality of life and worsening cancer prognosis. Multiple preclinical models, including the widely used mouse model of subcutaneous inoculation with the C26 colorectal carcinoma cell line, have been developed to study the biological mechanisms of CRC cachexia and elucidate potential new treatments. It has been proposed that a distinct cell line of the same origin, namely CT26, is relatively non-cachexic. However, studies evaluating the relative potential of C26 and CT26 cells to induce cancer cachexia in parallel have been limited. The differences in the biological mechanisms by which C26 and CT26 impact skeletal muscle mass and function have also not been fully elucidated. In the current study, we investigated the differential capacity of C26 and CT26 to induce cancer cachexia using both an *in vitro* cancer-muscle cell co-culture and an *in vivo* syngeneic mouse model. Our results show that both C26 and CT26 cells induced significant atrophy of murine C2C12 skeletal myotubes. In the mouse model, while C26 and CT26 both reduced skeletal muscle mass and fat mass, only C26 tumors led to loss of body weight and impaired skeletal muscle force output. We further show that C26 tumor-bearing mice exhibit greater muscle inflammation than CT26 tumor-bearing mice. In addition, mice bearing C26 and CT26 tumors showed differential regulation of the innate immune responses and muscle protein turnover. Overall, our data suggests that although both C26 and CT26 cells do exhibit cachexic effects, C26 cells induce greater loss in body weight, fat mass, skeletal muscle mass, and physical function via promoting chronic inflammation and deregulating protein balance of skeletal muscle.

## INTRODUCTION

Cancer cachexia is a multifactorial syndrome characterized by progressive loss of body weight, skeletal muscle mass, adipose tissue, and physical function (1). As the second leading cause of cancer-related mortality, colorectal cancer (CRC) is projected to impact over 150,000 members of the US population in 2026, with a growing burden of incidence in adults under 50 years old (2). Over 30% of CRC patients develop cancer cachexia, which is closely associated with cancer morbidity and mortality (3–5). During CRC progression, the loss of skeletal muscle mass is an independent predictor for worsened responses to anti-cancer treatments and poorer CRC prognosis (6, 7). However, despite active research on anti-cachexia interventions, medical strategies to alleviate cancer cachexia in CRC patients are currently limited (8).

Multiple preclinical models have been developed to investigate the biological mechanisms of CRC cachexia and discover potential novel anti-cachexic interventions. One of the most prevalent pre-clinical models of cachexia is that induced by colon-26 (C-26) colorectal carcinoma cells (9, 10). First established by Corbett et al. 1975, the C26 cell line was originally derived from female BALB/c mice receiving the colon carcinogen N-nitroso-N-methylurethane (NMU) (11). C26 tumors were identified as an undifferentiated colon carcinoma that was highly tumorigenic (11, 12). Extensive subsequent research has shown that tumor-derived factors secreted from C26 cells result in profound muscle wasting in the terminally differentiated cultures of the C2C12 murine skeletal muscle cell line (13, 14). C26 cells have also been widely adopted in animal models, with various studies testing subcutaneous, intravenous, or intramuscular injection induces tumor growth and cancer cachexia in both BALB/c and CD2F1 mice (10, 15–20). Early tumorigenicity research showed that injection of a dose of 1×10^5^ of C26 cells led to a 100% tumor induction by two weeks post-inoculation (12). Mice bearing C26 tumors display decreased body weight, skeletal muscle mass, and adipose tissue mass, as well as hypoglycemia and hepatic dysfunction (10, 21). Mechanistically, cachexia in C26 tumor-bearing mice has been associated with increased muscle inflammation, impaired anabolism/increased catabolism, disturbed membrane integrity, and impaired the mitochondria function (18, 22–25).

Another CRC cell line of the same origin, termed CT26, has been relatively less studied. The CT26 cell line was also originally derived from NMU-induced colon tumor in BALB/c mice, yet there is no record showing when and how the CT26 cell line diverged from C26 cells, and some studies even use the terms C26 and CT26 interchangeably (26). Recent research on tumor immunogenicity of mice bearing C26 versus CT26 carcinomas demonstrates distinct differences in genetic mutations and tumor immune infiltration of the two cell lines. While both cell lines have KrasG12D mutation, genomic profile demonstrates that CT26 cells possess unique mutations in oncogenes (e.g., *Birc5* and *Mki67*) and tumor suppressors (e.g., *Cdkn2a*) not observed in C26 cells (27, 28). On the other hand, C26 cells have a higher level of Wnt signaling activity than CT26 cells (29). Furthermore, *in vivo* studies have demonstrated that while CT26 tumor tissues are infiltrated with more cytotoxic T cells, dendritic cells, and natural killer (NK) cells, that C26 tumors rather exhibit lower T cell infiltration and higher resistance to anti-PD1 therapy (29).

Several studies have reported body weight loss and muscle wasting in CT26 tumor-bearing mice (30–33). Nevertheless, other groups have proposed that CT26 cells might be relatively incapable of inducing cancer cachexia (34–36). The inconsistent observations of investigators employing the CT26 model could perhaps be attributed to the differences in sources of CT26 and the physiological characteristics of the hosts. In addition, tumor progression and the efficacy of inducing cachexia could potentially be impacted by the site of tumor inoculation (37, 38). However, research directly comparing C26 and CT26 cells in parallel for their potential of inducing cancer cachexia and possible differences in biological mechanisms has been limited to date (34, 36).

It is worth mentioning that most prior C26 or CT26 inoculation studies were done in young mice of 6–8 weeks (39, 40). At this age, mice could still undergo increase in skeletal muscle fiber size and gain body weight (41, 42). Although prior research has shown that C26 successfully induces cancer cachexia in both young developing mice (8 weeks) and middle aged adult mice (12 months), differential impacts on fiber size and proteolysis pathways was observed between age groups (43). Notably, the average onset age of CRC in human populations is over 50 years old, with an increase in early-onset CRC (35–49 years) (44). Therefore, cachexia models using young mice of 6–8 weeks might not fully represent the physiological characteristics in adults developing CRC cachexia. Thus, it might be meaningful to investigate how CRC cachexia influences skeletal muscles in older adult mice that better represent the average onset of colorectal cancer in the human population (45, 46).

In the current study, we aimed to investigate the differential impacts of C26 and CT26 colon carcinoma cells on skeletal muscle physiology as well as the underlying biological mechanisms. To examine possible difference between *in vitro* and *in vivo* models of cancer cachexia, we used both a cancer-muscle cell co-culture model and a mouse model of subcutaneous tumor inoculation. While both C26 and CT26 cells induced comparable atrophy in C2C12 myotubes *in vitro*, C26 cell inoculation resulted in relatively greater decrements in skeletal muscle mass and strength in the BALB/c mice. Immunohistochemistry and molecular analysis revealed that C26 tumor-bearing mice exhibited greater skeletal muscle inflammation associated with an overexpression of the pro-inflammatory enzyme 5-lipoxygenase (5-LOX) and a marked depletion of the anti-inflammatory/pro-resolving enzyme 12/15-lipoxygenase (12/15-LOX). C26 and CT26 tumors also showed differential regulation of the muscle protein synthesis and degradation pathways.

## METHODOLOGY

### Cell culture:

The murine skeletal muscle cell line C2C12 (ATCC, CRL-1772) and two colorectal carcinoma cell lines including C26 (a gift from Dr. Keehong Kim at Purdue University) and CT26 (ATCC, CRL-2638) were cultured separately in Dulbecco’s modified Eagle medium (DMEM, Gibco, 11995–065) containing 10% fetal bovine serum (FBS, Corning, 35–010-CV) and antibiotics (penicillin 100 U/mL, streptomycin 100 μg/mL, Gibco, 15140–122) at 37 °C in a cell incubator with 5% CO_2_.

### Co-culture model:

When reaching a confluency of 70%, C2C12 cells were passaged and seeded into a 24-well co-culture base plate (Corning, 353504) in growth media at a cell density of 2.5 × 10^4^/cm^2^ and allowed to proliferate and crowd. Four days after plating, C2C12 myoblasts were induced to form myotubes in differentiation media (DM), DMEM containing 2% horse serum (HS, Gibco 26050088) and antibiotics as described above. C26 and CT26 cells were seeded in DM into the upper compartments of co-culture plate inserts (Corning, 353095) at a low density (4.17 × 10^4^/cm^2^), a moderate density (8.3 × 10^4^/cm^2^), or a high density (1.67 × 10^5^/cm^2^). Additionally, 8.3 × 10^4^/cm^2^ of C2C12 cells were plated in the inserts as a control for the co-culture experiment. 72 hours after C2C12 cells were induced to differentiate, the upper co-culture inserts were moved to the lower compartments of the base plates. Fresh DM was added to both C2C12 myotubes in the base plates and all the cells in the inserts when the co-cultures were established. C2C12 myotubes were co-cultured with C2C12 control, C26, or CT26 cells for 72 hours before fixation and immunocytochemistry as described below.

### Mice and tumor inoculation:

10-month-old female BALB/c mice were obtained from Charles River Laboratories and housed under specific pathogen-free conditions with ad libitum access to food and water. After one week of acclimatization, the mice were randomized to three groups with balanced starting body weights. Prior to tumor inoculation, C26 cells and CT26 cells were harvested separately and resuspended in sterile saline. Two groups of mice were inoculated with either C26 cells (5 × 10^5^ cells/100 μL saline per mouse, n=6) or CT26 cells (5 × 10^5^ cells/100 μL saline per mouse, n=7) subcutaneously in their upper right flank. Age- and sex-matched healthy control mice (n=6) were injected with sterile saline (100 μL/mouse). Mouse condition and body weight were monitored at least three times a week, and tumor dimensions (length and width) were measured by a caliper every other day starting Day 5 post inoculation. The animal study was approved by Purdue University IACUC protocol #2205002271.

### Measurement of body composition:

One day prior to euthanasia, the body composition of all the mice was scanned by magnetic resonance-based analysis (EchoMRI 3-in-1). Unanesthetized mice were individually placed in a plastic cylinder, which was then inserted into the scanner to measure body fat, lean mass, free water mass, and total water mass.

### Muscle force testing:

One day prior to euthanasia, mice were anesthetized with 2% isoflurane and placed on a platform heated to 37°C. The function of the ankle plantar flexor muscle group including the gastrocnemius (GAST), plantaris (PLA), and soleus (SOL) muscles were assessed by the non-invasive *in vivo* assay (1300A 3-in-1 Whole Animal System for Mice, Aurora Scientific). The knee joint of the right leg was immobilized by the knee clamp, and the foot was taped to the foot petal. Electrodes were placed superficially under the skin overlaying the GAST, and the muscle was stimulated after adjusting to optimal current to measure maximum isometric torque. To obtain the force-frequency relationship, the muscle was then stimulated at increasing frequencies (10, 25, 50, 80, 100, 150, 250, and 300 Hz) with a 1-minute rest between two stimulations. The mice were allowed to recover overnight after the *in vivo* test. On the day of euthanasia, mice were put under isoflurane anesthesia, and the distal part of the tibialis anterior (TA) muscle was isolated through dissection. During the *in situ* force test, the knee joint was pinned to the limb plate, and the distal tendon of TA was tied to the lever arm of the force-test apparatus by a 4/0 silk suture (FST, 18020–40). The TA muscle was stimulated with the platinum electrodes inserted within the overlying skin and fascia. Current and muscle length were adjusted to obtain the maximum isometric twitch force (P_t_) at optimal muscle length (L_o_). PBS was continuously applied to the exposed TA muscle. The muscle was then stimulated at increasing frequencies while held at L_o_ until reaching maximum isometric tetanic force (P_o_). Then, TA muscle was stimulated at increasing frequencies (10, 25, 50, 80, 100, 150, 250, and 300 Hz) and a 1-minute rest was allowed before each contraction. TA optimum fiber length (L_f_) was determined by Lo multiplying the TA muscle L_f_/L_o_ ratio of 0.6 (47). The CSA of the TA muscle was calculated as muscle mass divided by the product of L_f_ and 1.06 mg/mm^3^ (48). TA-specific tetanus force *sPo* was calculated as Po/muscle CSA.

### Muscle tissue collection:

After *in situ* force testing of the TA muscle, all the animals were euthanized via cervical dislocation and bilateral pneumothorax while under isoflurane anesthesia, and hindlimb muscles including GAST, PLA, SOL, TA, and extensor digitorum longus (EDL)from both legs were dissected and weighed. Whole muscles from the left leg and the proximal half of the right leg muscles were snap frozen in liquid nitrogen for RNA and protein extraction, and the distal half of the muscles were embedded in Optimal Cutting Temperature (O.C.T.) compound and frozen in isopentane cooled on liquid nitrogen. All the samples were stored in −80°C freezer until downstream analysis.

### Immunocytochemistry:

To assess cell morphology in the co-culture model, C2C12 myotubes were fixed in 4% paraformaldehyde (PFA, Electron Microscopy Sciences, 15710) for 30 minutes at 4°C. The cells were then permeabilized with 0.1% Triton X-100 for 30 minutes and blocked in 1% bovine serum albumin (BSA, Sigma-Aldrich A3294–50G) for 1 hour at room temperature, followed by overnight incubation with primary antibodies against sarcomeric myosin (MF20c, DSHB, 1:100) and myogenin (F5Dc, DSHB, 1:100) at 4°C. The following day, C2C12 cells were washed in PBS and then incubated with secondary antibodies including Goat Anti-Mouse IgG1 Alexa Fluor 568 (Invitrogen A-21124, Thermo Fisher Scientific, 1:500) and Goat Anti-Mouse IgG2b Alexa Fluor 647 (Invitrogen, Thermo Fisher Scientific A-21242, 1:500). DAPI (Invitrogen, Thermo Fisher Scientific D21490, 2 μg/mL) was used to counterstain cell nuclei. C2C12 myotubes were visualized using a fluorescent microscope (Echo Revolution) operating in inverted configuration, and fluorescent images were captured at a magnification of 10×. Nine images were automatically captured from the same predetermined positions in each culture well to avoid investigator bias.

### Immunohistochemistry:

10 μm cryosections were cut from OCT embedded SOL and EDL muscle midbelly in a cryostat at −20°C. Sections were adhered to SuperFrost Plus slides and air-dried. Unfixed slides were used for Hematoxylin & Eosin (H & E) and fiber type staining. For immune cell staining, slides were fixed in acetone at −20°C for 10 minutes. All the slides used for immunostaining were blocked in M.O.M immunoglobulin blocking reagent (Vector Laboratories, BMK-2202) for 1 hour at room temperature before overnight incubation at 4°C with primary antibodies. The following day, slides were washed in PBS solution and incubated with secondary antibodies for 1 hour at room temperature. After washing in PBS, the slides were mounted using Fluorescence Mounting Medium (Agilent Dako, S3023) and fluorescent images were captured using Echo Revolution microscope operating in upright configuration. Primary antibodies were used to stain MyHC I (DSHB, BA-D5c, 1:100), MyHC IIa (DSHB, SC-71c, 1:100), MyHC IIb (DSHB, BF-F3c, 1:100), laminin (Abcam, ab7463, 1:100 or Novus Biologicals N-600–883, 1:100), Gr-1 (Bio-Rad, MCA2387, 1:50), CD68 (Bio-Rad, MCA1957, 1:100 or Abcam, ab283654), and CD206 (Bio-Rad, MCA2235, 1:100). Secondary antibodies used for corresponding primary antibodies included Goat Anti-Rabbit IgG (H+L) Alexa Fluor 350 conjugate (Invitrogen, Thermo Fisher Scientific, A-21068), Goat Anti-Rat IgG (H+L) Alexa Fluor 555 conjugate (Invitrogen, Thermo Fisher Scientific, A-21434), Goat Anti-Mouse IgG1 Alexa Fluor 488 conjugate (Invitrogen, Thermo Fisher Scientific, A-21221), and Goat Anti-Rabbit IgG (H+L) Alexa Fluor 647 conjugate (Invitrogen, Thermo Fisher Scientific, A-21244). DAPI (Invitrogen, Thermo Fisher Scientific, D21490, 2 μg/mL) was used to counterstain cell nuclei on the immune marker slides.

### Image analysis:

In the co-culture model, overall cell density, myotube area, percentage of myogenin^+^ cells, and myotube diameter were analyzed using ImageJ/FIJI as quantitative indices for myotube differentiation and growth. To determine mean myotube diameter, the ten largest myosin-positive cells from each image were manually assessed at their widest uniform point perpendicular to the length of the cell using the straight-line selection tool in the software. For branched myotubes, each branch was measured as a single myotube, and the region where the branches join excluded from the analysis. At least three images were analyzed for each biological replicate. Total cell number (DAPI^+^ cells/mm^2^), percentage of myogenin^+^ cells (myogenin^+^/DAPI^+^ cells) (%), and percentage of myosin-positive area (myotube area) (%) per field of view were quantified using a custom automated in-house plugin for ImageJ/FIJI. Muscle fiber typing images were analyzed and quantified using MuscleJ 1.0.2 with minor modifications (49). EDL and SOL samples were analyzed with customized fiber type detection sensitivity thresholds of SD=1 and SD=0.5, respectively. Immune cells including CD68^+^, CD206^+^, and Gr-1^+^ cells were manually counted throughout the entire EDL or SOL cross section, which was then normalized to total tissue area determined by entire mode of MuscleJ 2.0 (50).

### RNA extraction and RT-qPCR:

Tissue RNA from the frozen proximal half of the GAST muscle (~25 mg) was extracted using TRIzol Reagent (Invitrogen, ThermoFisher Scientific, 15596018). GAST tissue samples were homogenized using in the Mixer Mill MM 400 (Retsch) bead homogenizer for 10 minutes in safe-lock microtubes containing zirconium beads. Then, bromochloropropane was used for supernatant phase separation prior to RNA isolation with assistance with Direct-zol^™^ RNA MiniPrep Plus kit (Zymo Research, R2072), and on-column DNase treatment. RNA concentration and purity was assessed using the Nanodrop 2000c UV-Vis Spectrophotometer (Thermo Scientific). Following cDNA synthesis using High-Capacity cDNA Reverse Transcription Kit (Applied Biosystems, Thermo Fisher Scientific, 4368813), the level of mRNA was measured by reverse transcription quantitative polymerase chain reaction (RT-qPCR) using SYBR^™^ Green PCR Master Mix (Applied Biosystems, Thermo Fisher Scientific, 4312704). RT-qPCR plates were run using the CFX Opus 96 Real-Time PCR System (Bio-Rad Laboratories). Several reference genes were tested for expression stability. The mRNA expression of Valosin-containing protein (VCP, *Vcp*), ER membrane protein complex subunit 7 (EMC7, *Emc7*), and GAPDH (*Gapdh*) were identified as the least variable and were used as reference genes. The geometric mean of these three reference genes was used for normalization. The sequences of all the primers used are shown in Table 1. RT-PCR data were analyzed using the 2^−ΔΔCT^ method.

### Protein extraction and western blot:

Frozen GAST tissue from the left leg (~40 mg) was homogenized in ice-cold 1 × RIPA buffer (MilliporeSigma, 20188, 15 μL/mg tissue) with 1 × Halt Protease and Phosphatase Inhibitor Cocktail (Thermo Fisher Scientific, 78442) using a Fisherbrand Bead Mill 4 mini homogenizer (Thermo Fisher Scientific, 15–340-164) at 5m/s for 60 seconds. Tissue homogenates were incubated with mild agitation at 4°C for 1 hour. After centrifugation at 13000 × g for 10 minutes at 4°C, the supernatant containing protein was collected. Pierce bicinchoninic acid (BCA) protein assay kit (Thermo Fisher Scientific, PI23225) was used to determine protein concentration in each sample, and protein samples were diluted to 2 μg/μL in 1 × Laemmli buffer (Bio-rad, 1610737). The protein samples were boiled for 5 min, and an equal volume of protein (20 μg) was loaded to a 12% sodium dodecyl sulfate-polyacrylamide gel electrophoresis (SDS-PAGE). After separation by gel electrophoresis, the protein samples were transferred to a nitrocellulose membrane using a Trans-Blot Turbo Transfer System (Bio-Rad, 1704150). Membranes were blocked in 5% skim milk powder in Tris buffered saline with 0.1% Tween 20 (TBST) for 1 hour at room temperature. Then, membranes were incubated with primary antibodies diluted in 5% Bovine Serum Albumin (BSA) in TBST overnight at 4 °C with gentle agitation. Primary antibodies tested included Rabbit Anti-15-LOX-1 (Abcam, ab244205, 1:1,000), Rabbit Anti-S6 Ribosomal Protein (Cell Signaling Technology, 5G10, 1:1,000), Rabbit Anti-Phospho-S6 Ribosomal Protein (Ser235/236) (Cell Signaling Technology, 4858S, 1:1,000), Rabbit Anti-Phospho-S6 Ribosomal Protein (Ser240/244) (Cell Signaling Technology, 5364S, 1:1,000), Rabbit Anti-p70 S6 Kinase (Cell Signaling Technology, 2708, 1:1,000), Rabbit Anti-Phospho-p70 S6 Kinase (Thr421/Ser424) (Cell Signaling Technology, 9204S, 1:1,000), Rabbit Anti-p44/42 MAPK (Erk1/2) (Cell Signaling Technology, 9102, 1:1,000), and Rabbit Anti-Phospho-p44/42 MAPK (Erk1/2) (Thr202/Tyr204) (Cell Signaling Technology, 4370, 1:1,000). Mouse Anti-GAPDH was used to probe for the loading control (Santa Cruz Technology, sc-32233, 1:1,000). The following day, membranes were washed in TBST for 5 minutes × 3 times and probed with a Goat Anti-Rabbit IgG (H + L) Horseradish Peroxidase (HRP) conjugated secondary antibody (Jackson Labs, 111–035-144, 1:10,000) or Goat Anti-Mouse IgG (H + L) Horseradish Peroxidase (HRP) conjugated secondary antibody (Jackson Labs, 115–035-003, 1:10,000) diluted in 5% skim milk in TBST for 1 hour at room temperature. Protein bands were visualized using Clarity Western enhanced chemiluminescent (ECL) substrate (Bio-Rad, 1705060), and chemiluminescent signals were captured by a ChemiDoc Imaging System (Bio-Rad, 12003153). Densitometry analysis was performed using Image Lab 6.1 software (Bio-Rad).

### Statistical analysis:

Data are presented as the mean ± SEM, with raw data from each biological replicate displayed as dot plots on column graphs. Statistical analysis was performed in GraphPad Prism 10. In experiments with only one independent variable and three levels (i.e., Ctrl vs. CT26 vs. C26), differences between groups were tested by a one-way ANOVA followed by pairwise Fisher’s Least Significant Difference (LSD) post hoc tests. For experiments with two independent variables (e.g., time and cell type), between-group differences were tested by a two-way ANOVA (main effect of cell type) followed by pairwise Fisher’s Least Significant Difference (LSD) post hoc tests. P ≤ 0.05 was used to determine statistical significance. Statistical outliers were identified by Grubbs’ test with α=0.05, with at most one outlier removed from each group.

## RESULTS

### C26 and CT26 Exhibited Similar Potency in Inducing *In Vitro* Muscle Atrophy

**Figure 1.**
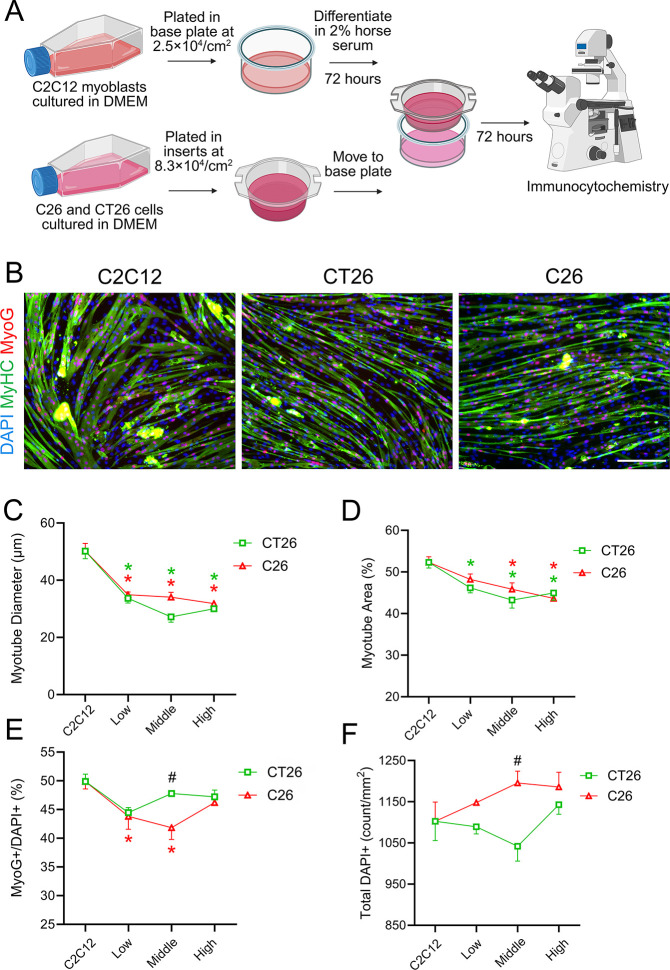
CT26 and C26 carcinoma cells induced *in vitro* muscle atrophy in a cancer-muscle co-culture model. **(A)** Subconfluent C2C12 myoblasts cultured in DMEM were plated in the 24-well base plate at 2.5 × 10^4^/cm^2^. C2C12 myoblasts proliferated for four days and were induced to differentiate in differentiation media (DM), DMEM containing 2% horse serum for 72 hours. On the same day of inducing differentiation, C26 cells and CT26 cells were plated in the co-culture inserts at increasing densities (4.17 × 10^4^/cm^2^, 8.3 × 10^4^/cm^2^, and 1.67 × 10^5^/cm^2^) and proliferated for 72 hours. C2C12 myoblasts were plated in the inserts at 8.3 × 10^4^/cm^2^ as a negative co-culture control. Then, the co-culture inserts containing C26, CT26, or C2C12 cells were moved to the lower compartments of the base plate. Fresh DM was added to both inserts and lower compartments. **(B)** The co-culture continued for 72 hours, and cells were fixed with 4% paraformaldehyde (PFA) and stained for myosin heavy chain (MyHC, green) and myogenin (MyoG, red). DAPI was used to counterstain the nuclei (blue). Scale bar is set at 200 μm. Myotube diameter **(C)**, percentage of myotube area **(D)**, MyoG^+^/total DAPI^+^ percentage **(E)**, and total DAPI^+^ cells **(F)** were quantified to assess potential atrophic effect of C26 cells and CT26 cells. *P<0.05 for pairwise comparison to C2C12 insert control, #P<0.05 for comparison between C26 and CT26 cells.

To investigate the effects of soluble factors secreted by C26 and CT26 carcinoma cells in inducing skeletal muscle cell wasting *in vitro*, we co-cultured mature C2C12 myotubes with increasing densities of C26 or CT26 cells (Low: 4.17 × 10^4^/cm^2^, Middle: 8.3 × 10^4^/cm^2^, and High: 1.67 × 10^5^/cm^2^) to allow for continuous exchange of muscle- and cancer-secreted factors (Figure 1A). Immunocytochemistry analysis showed that the 72-hour co-culture with either C26 or CT26 cells induced a significant atrophy of C2C12 myotubes (Figure 1B). Both C26 and CT26 cells led to a marked decrease in C2C12 myotube diameter (Figure 1C). In addition, C26 and CT26 cells both reduced the percentage of myotube area at middle and high densities (Figure 1D). While CT26 cells did not influence myogenin expression, C2C12 myotubes co-cultured with C26 cells had a reduced proportion of myogenin^+^ cells at low and middle cancer cell co-culture densities (Figure 1E). There was no difference in cell viability when compared to control as indicated by the total number of DAPI^+^ cells/mm^2^ at any cell density (Figure 1F). Overall, these data show that both CT26 and C26 carcinoma cells induce robust *in vitro* skeletal muscle cell wasting.

### C26 Induced a Larger Loss in Body Weight, Lean Mass, and Fat Mass

**Figure 2.**
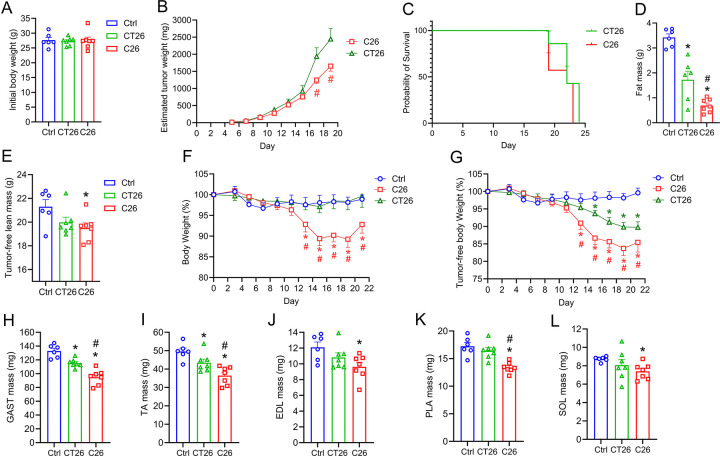
C26 and CT26 tumor inoculation induced changes in body weight and body composition. **(A)** Female BALB/c mice were randomized to three groups with similar initial body weight and injected with saline (n=6), 5 × 10^4^ CT26 cells (n=7), or 5 × 10^4^ C26 cells (n=6) subcutaneously into the right flank. **(B)** Tumor diameters were measured every other day and estimated tumor weight was calculated as 0.52 × length × width^2^. Probability of survival curve was plotted based on mice being euthanized after meeting the humane endpoint criteria **(C)**. One day prior to euthanasia, EchoMRI was used to assess body composition including lean mass, free water, and fat mass **(D)**. Tumor-free lean mass **(E)** was calculated as absolute lean mass minus endpoint tumor mass. Total body weight (F) was assessed every other day and tumor-free body weight was calculated as total body weight subtracted by the estimated tumor weight **(G)**. The hindlimb skeletal muscles were weighed after dissection, including gastrocnemius (GAST) **(H)**, tibialis anterior (TA) **(I)**, extensor digitorum longus (EDL) **(J)**, plantaris (PLA) **(K)**, and soleus (SOL) **(L)**. *P<0.05 for difference compared to healthy controls, #P<0.05 for difference between C26 and CT26 tumor-bearing mice.

To study the potential of C26 and CT26 tumors in inducing cancer cachexia *in vivo*, three groups of female BALB/c mice with the same initial body weight were subcutaneously injected with saline (Ctrl), C26 cells (5×10^5^ cells/mouse), or CT26 cells (5×10^5^ cells/mouse) at the right flank (Figure 2A). Tumors formed C26 and CT26 cells became palpable and measurable 5 days after inoculation. Interestingly, mice inoculated with CT26 cells developed larger subcutaneous tumors compared to those inoculated with C26 cells despite a similar probability of survival (Figure 2B and C). EchoMRI analysis of body composition revealed that both C26 and CT26 tumors induced fat wasting, and C26 tumor-bearing mice experienced a higher loss of fat mass than CT26 mice (Figure 2D). On the other hand, only C26 tumors resulted in a significantly lower tumor-free lean mass at endpoint (Figure 2E). In addition, while C26 tumor-bearing mice experienced a significant loss of total body weight starting day 13 post inoculation, CT26 tumor-bearing mice did not exhibit any such loss in total body weight (Figure 2F). Nevertheless, both C26 and CT26 tumors led to a marked reduction in estimated tumor-free body weight, although C26 tumors exhibited both an earlier onset and greater magnitude of weight loss than CT26 mice (Figure 2G). Both C26 and CT26 tumors caused a significant loss of absolute muscle mass in GAST and TA, with C26 resulting in more muscle wasting (Figure 2H and I). However, in smaller hindlimb muscles including PLA, EDL, and SOL, only C26 tumors induced a significant loss of muscle mass compared to healthy controls (Figure 2J–L).

### C26 Tumors Induce Greater Skeletal Muscle Wasting Compared to CT26

**Figure 3.**
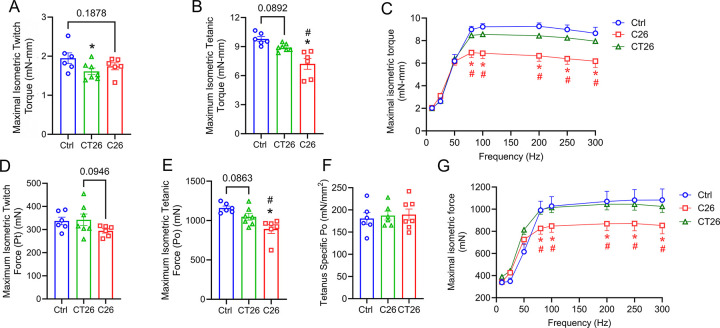
C26 and CT26 tumors differentially impacted the muscle mass and force output of BALB/c mice. To determine the force output of gastrocnemius muscle (GAST), maximal isometric twitch torque **(A)** and maximal isometric tetanic torque **(B)** were measured by *in vivo* force test one day prior to euthanasia. The force-frequency curve of GAST muscle was generated by muscle stimulation at increasing electric frequencies (10, 25, 50, 80, 100, 150, 250, 300 Hz) with a 1-minute rest between contractions **(C)**. After overnight recovery, the force output of tibialis anterior (TA) was assessed by maximal isometric twitch force **(D)** and maximal isometric tetanic force **(E).** TA-specific tetanus force was calculated as described in method **(F)**, and the force-frequency curve of TA was generated similar to that of GAST **(G)**. *P<0.05 for difference compared to healthy controls, #P<0.05 for difference between C26 and CT26 tumor-bearing mice.

Next, we investigated the influence of C26 and CT26 tumors on skeletal muscle function and mass. To test whether tumor progression affects GAST function, all mice were tested for the maximal plantar flexion torque one day prior to euthanasia. CT26 tumors decreased the single twitch torque of GAST muscle and had a trend to reduce the absolute peak torque during tetanus (Figure 3A and B). On the other hand, while C26 tumor did not significantly reduce the twitch torque, it induced a greater reduction of maximal tetanic torque compared to CT26 tumor-bearing mice (Figure 3A and B). Moreover, C26 tumors led to a decrease in maximal isometric torque when stimulated by medium to high frequencies (80 Hz~300 Hz), while no difference was detected in CT26 tumor-bearing mice when increasing frequencies were applied to GAST (Figure 3C).

To assess the potential change in the muscle function of TA, the hindlimb muscle primarily responsible for ankle dorsiflexion, we performed the *in situ* force test on TA muscle after overnight recovery following the GAST force test. C26 tumor-bearing mice tended to have a reduced isometric twitch force (Figure 3D). Like in GAST, CT26 tended to reduce maximal tetanic force, but C26 tumors resulted in a considerably greater reduction in TA tetanus force (Figure 3E). However, no difference was observed in TA-specific tetanus force produced by the healthy controls and the tumor-bearing mice (Figure 3F). When the TA was stimulated at increasing frequencies, only C26 tumor-bearing mice displayed a decreased isometric force production compared to healthy controls at stimulation between 80 Hz to 300 Hz (Figure 3G).

### C26 and CT26 Tumors Resulted in Changes in Skeletal Muscles Fiber Morphology

**Figure 4.**
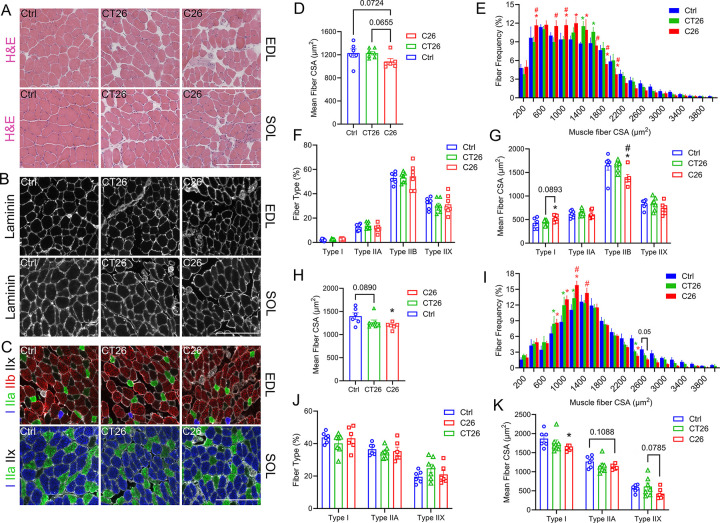
C26 and CT26 tumors resulted in morphological changes in fast-twitch and slow-twitch muscles. **(A)** Overall tissue structures of extensor digitorum longus (EDL) and soleus (SOL) from healthy controls, C26 tumor-bearing mice, and CT26 tumor-bearing mice were visualized by hematoxylin and eosin (H&E) staining. **(B)** The muscle basement membranes of EDL and SOL were visualized by immunostaining for laminin (white). **(C)** Different fiber types were identified in EDL and SOL by immunofluorescence. Myosin heavy chain I (MHC I) fibers were stained by BA-D5c (blue), MHC IIa was stained by SC-71c (green), and MHC IIb was stained by BF-F3c (red). Scale bar is set at 200 μm. Fiber morphology of EDL muscles was assessed by mean cross-sectional area (CSA) **(D)**, fiber size distribution frequency **(E)**, fiber type percentage **(F)**, and fiber type-specific mean CSA **(G)**. SOL fiber morphology was also determined by mean CSA **(H)**, fiber size distribution frequency **(I)**, fiber type percentage **(J)**, and fiber type-specific mean CSA **(K).** *P<0.05 for difference compared to healthy controls, #P<0.05 for difference between C26 and CT26 tumor-bearing mice.

To better characterize how C26 and CT26 tumors influence different muscle types, we investigated the changes in muscle morphology using the EDL and SOL as representative types of fast-twitch and slow-twitch muscles, respectively (Figure 4A–C). Immunohistochemistry staining shows that C26 tumors reduced mean myofiber cross-sectional area (CSA) of the SOL and tended to decrease mean fiber of the EDL (Figure 4D and H). Although not statistically different, CT26 also showed a trend to decrease the CSA of both EDL and SOL (Figure 4D and H). The fiber frequency distribution of SOL and EDL further showed that C26 tumor-bearing mice exhibited a shift towards smaller myofibers (Figure 4E and I), yet we did not observe any change in percentage of fiber type composition in either C26 or CT26 tumor-bearing mice muscles (Figure 4F and J). The atrophic effect of C26 tumors in EDL was predominantly caused by smaller type IIb fibers, while the type I fibers are rather larger in EDL muscles of C26 tumor-bearing mice (Figure 4G). Intriguingly, the decrease in muscle fiber size in the SOL of C26 tumor-bearing mice was mainly driven by the atrophy of type I slow-twitch muscle fibers (Figure 4K). However, no significant difference was detected in fiber-specific CSA in either EDL (Figure 4G) or SOL (Figure 4K) of CT26 tumor-bearing mice. Overall, C26 tumor development resulted in a higher level of muscle atrophy in SOL and EDL.

### Differential Intramuscular Immune Responses of C26 and CT26 Tumor-bearing Mice

**Figure 5.**
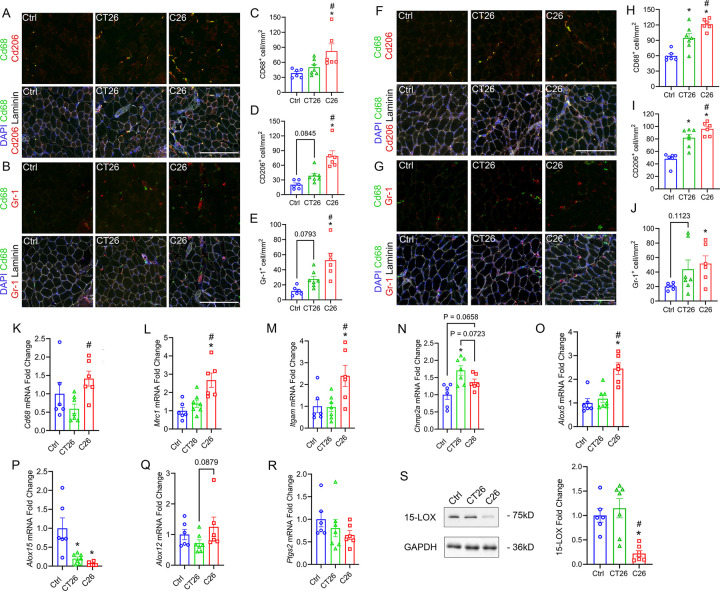
C26 tumor-bearing mice exert a higher level of intramuscular immune response than CT26 tumor-bearing mice. Sections of EDL **(A-B)** and SOL **(F-G)** were stained for immune cells including macrophages (MΦ) (CD68^+^ cells), M2-like MΦ (CD206^+^ cells), and polymorphonuclear neutrophils (PMNs) (Gr-1^+^ cells). Intramuscular immune infiltration was assessed by the quantification of CD68^+^ cells, CD68^+^CD206^+^ cells, and Gr-1^+^ cells in EDL **(C-E)** and SOL **(H-J)**. Scale bar is set at 200 μm. The mRNA expression level of *Cd68* **(K)**, *Mrc-1* **(L),** *Itgam* **(M)**, *Chmp2a* **(N)**, *Alox5* **(O)**, *Alox15* **(P),** *Alox12* **(Q),** and *Ptgs2* **(R)** was determined by real-time quantitative reverse transcription PCR (RT-qPCR). Gene expression was normalized to the geometric mean of *Vcp*, *Emc7*, and *Gapdh*. Western blot was used to examine the protein expression of leukocyte-type 12/15-LOX **(S)**. *P<0.05 for difference compared to healthy controls, #P<0.05 for difference between C26 and CT26 tumor-bearing mice.

To determine immune cell infiltration of skeletal muscles during cancer cachexia, total macrophages (MΦ) (CD68^+^ cells), M2-like MΦ (CD68^+^CD206^+^ cells), and polymorphonuclear neutrophils (PMNs, Gr-1^+^ cells) were stained in EDL and SOL (Figure 5A–B, and F–G). In the EDL, while C26 tumor-bearing mice had a higher number of MΦ, CT26 tumors did not induce any significant increase in MΦ infiltration (Figure 5C). Moreover, more M2 MΦ were found in EDL of C26 tumor-bearing mice compared to CT26 tumor-bearing mice (Figure 5D). C26 also induced a significantly higher number of intramuscular PMNs, and there was a trend for CT26 to increase PMN infiltration compared to healthy controls (Figure 5E). On the other hand, in SOL, both C26 and CT26 tumors resulted in greater intramuscular infiltration of total MΦ and M2 MΦ compared to healthy controls, with C26 tumor-bearing mice showing a higher level of MΦ infiltration (Figure 5F, H and I). Moreover, C26 tumor-bearing mice induced intramuscular infiltration of PMNs and CT26 tended to increase PMN infiltration in SOL (Figure 5G and J).

To study the transcriptional regulation of inflammatory responses in skeletal muscles, we assessed the mRNA expression levels of innate immune markers in GAST muscle including Cluster of Differentiation 68 (CD68, *Cd68*), CD206 (*Mrc-1*), and CD11b (*Itgam*). While *Cd68* expression was not different between CT26 tumor-bearing mice and healthy controls, C26 tumors induced a significant increase in *Cd68* expression (Figure 5K). Meanwhile, we observed a significant increase in *Mrc-1* and *Itgam* expression in C26-bearing mice but not CT26-bearing mice (Figure 5L and M). We also measured the expression of Charged Multivesicular Body Protein 2A (CHMP2A, *Chmp2a*), which regulates autophagy and mediates the cytotoxicity of natural killer (NK) cells in tumor immunity (45, 46). We found that CT26 tumors induced an increase in *Chmp2a* expression, and C26 tumors had a trend to increase the level of *Chmp2a* (Figure 5N).

We also measured the expression of major pro-inflammatory cytokines including Monocyte Chemoattractant Protein-1 (MCP-1, *Ccl2*), Tumor Necrosis Factor-alpha (TNFα, *Tnf*), interleukin-6 (IL-6, *Il6*), and interleukin-1 beta (IL-1β, *Il1b*). Intriguingly, C26 did not change the expression of *Ccl2* and *Tnf* while CT26 tumors repressed the expression of *Ccl2* and *Tnf* in GAST muscle (Figure S1A and B). Moreover, there was no difference in the expression of *Il6* and *Il1b* in either C26 or CT26 tumor-bearing mice (Figure S1C and D). Additionally, there was no change in the expression of External Transcribed Spacer (ETS, *ETS*) transcription factors or Internal Transcribed Spacer-5.8S ribosomal DNA region (ITS-5.8S, *ITS-5.8S*), which is involved in skeletal muscle ribosome biogenesis (Figure S1E and F) (47).

We then assessed the expression of key enzymes involved in the biosynthesis of lipid mediators and immune regulation, including 5-lipoxygenase (5-LOX, *Alox15*), leukocyte 12/15-LOX (12/15-LOX, *Alox15*), platelet 12-LOX (12-LOX, *Alox12*), and cyclooxygenase 2 (COX-2, *Ptgs2*). We found that C26 tumors significantly increased the expression *Alox5*, which was not observed in CT26 tumors (Figure 5O). Interestingly, both C26 and CT26 tumors greatly diminished the expression of *Alox15*, which plays an important role in the production of specialized pro-resolving mediators (SPMs) and the resolution of muscle inflammation (48, 49) (Figure 5P). Further analysis on 15-LOX expression by western blot confirmed the robust downregulation at the protein level in C26 tumor-bearing mice, but 15-LOX protein was not reduced in CT26 mice (Figure 5S). COX-2 and 12-LOX have been shown to promote CRC cachexia through activating inflammation and repressing protein synthesis pathways (32, 50). Nevertheless, the expression of *Alox12* or *Ptgs2* in GAST muscles was not impacted by either C26 or CT26 (Figure 5Q and R).

### C26 and CT26 Modulate Skeletal Muscle Protein Turnover Pathways

**Figure 6.**
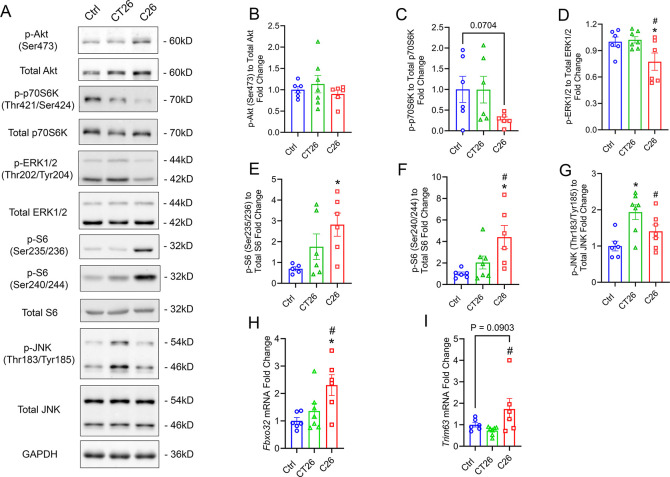
C26 and CT26 tumors exert different impacts on muscle protein synthesis and degradation pathways. **(A)** Protein samples were extracted from GAST muscles, and western blot was used to determine the phosphorylation levels of Akt (Ser473) **(B)**, p70S6K (Thr421/Ser414) **(C)**, ERK1/2 (Thr202/Tyr204) **(D)**, S6 (Ser235/236) **(E),** S6 (Ser240/244) **(F)**, and JNK (Thr183/Tyr185) **(G)**. GAPDH was used as a loading control. The mRNA expression level of the muscle-specific E3 ubiquitin ligases Atrogin-1 (*Fbxo32*) **(H)** and MuRF1 (*Trim63*) **(I)** were measured by RT-qPCR. Gene expression was normalized to the geometric mean of *Vcp*, *Emc7*, and *Gapdh*. *P<0.05 for difference compared to healthy controls, #P<0.05 for difference between C26 and CT26 tumor-bearing mice.

To study the influence of C26 and CT26 tumors on regulating muscle protein turnover pathways, we determined the phosphorylation levels of key enzymes involved in protein synthesis signaling in GAST muscles (Figure 6A). Neither C26 nor CT26 tumor inoculation changed the phosphorylation level of protein kinase B (Akt) at Ser473 (Figure 6B). While CT26 tumors did not influence the phosphorylation level of p70 ribosomal protein S6 kinase (p70S6K) (Thr421/Ser424), C26 tumors showed a trend in decreasing the phosphorylation level of p70S6K (Figure 6C). We also observed a slight yet significant decrease in the phosphorylation level of Extracellular Signal-Regulated Kinase 1/2 (ERK1/2, Thr202/Tyr204) (Figure 6D). Intriguingly, there was a significant increase in the phosphorylation level of ribosomal protein S6 (S6) at both Ser235/236 and Ser240/244 sites in C26 tumor-bearing mice (Figure 6E and F). We also observed an increase in the phosphorylation level of the stress-activated protein kinase (SAPK) c-Jun N-terminal kinase (JNK) (Thr193/Tyr185) in CT26 but not C26 tumor-bearing mice.

In addition, the mRNA expression level of the biomarkers for protein degradation and muscle atrophy in GAST was measured by RT-qPCR, including the muscle-specific ubiquitin ligases Atrogin-1 (*Fbxo32*) and MuRF1 (*Trim63*). There was a significant upregulation of *Fbxo32* in C26 GAST but not CT26 tumors (Figure 6F). Similarly, C26 tended to induce an increase in *Trim63*, while CT26 tumors did not change the expression of *Trim63* (Figure 6G).

## DISCUSSION

Colorectal cancer (CRC) cachexia involves involuntary loss of skeletal muscle mass and function, which could lower quality of life in cancer patients and reduce overall prognosis (1, 51). Although both the C26 and CT26 murine colon carcinoma cell lines have been used to model CRC cachexia, limited research has directly compared their relative potential to induce cancer cachexia. In the current study, we investigated the capacity of C26 and CT26 to induce CRC-associated cachexia in both *in vitro* and *in vivo* models. While both cell lines resulted in muscle atrophy *in vitro*, C26 tumors exhibited a higher capacity of inducing cachexia in a mouse model. We also show that C26 and CT26 could influence skeletal muscle dynamics through different biological mechanisms.

Previous research demonstrates that conditioned media (CM) from both C26 and CT26 carcinoma cells induces significant atrophy of murine C2C12 skeletal myotubes (52–55). A recent study used a co-culture model to investigate the crosstalk between C26 cells and C2C12 cells, showing that co-culture with C26 cells led to severe muscle atrophy (14). Using the muscle-cancer cell co-culture model, we tested the relative potency of C26 and CT26 cells to induce C2C12 atrophy at different seeding densities. We found that CT26 and C26 cells equivalently induced muscle atrophy in C2C12 myotubes, indicated by a significant decrease in myotube diameter. While CT26 cells led to a higher reduction in total myotube area, only C26 decreased the myogenin/DAPI ratio. As a crucial myogenic regulatory factor, myogenin drives the terminal differentiation of myoblasts and is critical for the expression of late muscle genes including muscle creatine kinase (MCK) (56). It has been shown that dysfunctional myogenin interferes with myocyte fusion and leads to smaller fiber size *in vivo* (57). In addition to regulating muscle differentiation, myogenin also plays important roles in maintaining adult muscle fibers and regulating muscle stem cells (58). Our results indicate that while both C26 and CT26 cells induced muscle atrophy, C26 cells further impaired myogenin expression in mature C2C12 myotubes, which could contribute to additional defects in myotube fusion and maintenance.

On the other hand, C26 and CT26 cells exhibited differential capacity to induce cachexia in the mouse model. Although both CT26 and C26 tumor-bearing mice had a reduced tumor-free body weight, C26 tumors led to a higher loss of tumor-free lean mass and skeletal muscle mass. Intriguingly, contrary to prior research, we show that both C26 and CT26 tumors led to reduced weight in GAST and TA, while a decrease in PLA, EDL, and SOL mass was only observed in C26 tumor-bearing mice (34). Differential changes of skeletal muscle mass are observed in different tumor models, and a higher reduction in muscle mass often indicates a more severe or late-stage cachexia (59, 60). In addition, we found that C26 tumors resulted in more severe muscle dysfunction. Consistent with previous research using animal models of cancer cachexia, C26 tumor-bearing mice in our study showed a deficit in force output coupled with a significant decrease in muscle mass (59, 61, 62). Despite the observed decrease in muscle mass of TA and GAST, CT26 tumor-bearing mice did not exhibit loss of TA or GAST strength during force frequency testing. Taken together, the reduced muscle mass yet preserved contractility in CT26 tumor-bearing mice could indicate a milder or early-stage cancer cachexia compared to C26 tumors.

In the current study, we also observed muscle-specific changes in fiber morphology and composition of C26 tumor-bearing mice. Previous research suggests that cancer progression differentially affects fast-twitch and slow-twitch fibers (63). Muscles rich in oxidative type I muscle fibers have been proposed to be more resistant to cancer-related atrophy than muscles predominantly composed of fast-twitch type IIb and IIx fibers, which could be associated with fiber-type-specific metabolic dysfunction (64, 65). For example, prior research shows that C26 tumor progression largely reduced TA fiber area but did not cause any significant changes in SOL fiber CSA (66). There has been other research reporting that C26 tumors led to muscle atrophy and reduced force output in SOL, yet the decrease in the mean fiber CSA was primarily driven by atrophy of relatively glycolytic type IIa and type IIb/IIx fibers (67). However, our model shows that C26 tumors induced a decrease in SOL mean myofiber CSA, and such decrease was driven mainly by the reduced mean CSA of type I fibers. Unlike prior research in C26 tumor bearing mice, we did not observe fiber-type switch from type I to type IIa or type IIx/b fibers in SOL (67). In the fast-twitch muscle EDL, we observed that C26 tumors had a strong trend in reducing mean CSA, although this was not statistically significant. Previous research shows that C26 tumors reduced the mean CSA in both type IIa and type IIb/IIx fibers within the EDL of male CD2F1 mice (67). On the contrary, we did not see any decrease in the mean CSA of type IIa or type IIx fibers in EDL of C26 tumor-bearing mice. Instead, the atrophic effect was mainly driven by reduced CSA of type IIb fibers. Notably, we also observed an increase in type I fiber CSA in the EDL of C26 mice. Previous research has reported a decrease in percentage of type IIa fiber and an increase in percentage of type IIb/IIx fiber in EDL (67). Nevertheless, no difference was seen in the percentage of fiber types in EDL in our C26 model. These inconsistent findings in SOL and EDL fiber composition could be related to sex difference, mouse strain, mouse age, and protocols for fiber type analysis.

In addition to muscle mass, C26 tumor-bearing mice showed a higher reduction in fat mass compared to CT26 tumor-bearing mice. The decrease in adipose tissue in cancer cachexia could be attributed to increased lipolysis, white adipose tissue browning, and altered metabolism featured by higher energy expenditure (68–70). Previous research demonstrates a rewiring of adipose metabolism in C26-induced cachexia, upregulating the production of metabolites including sarcosine and thymidine (71). It has also been shown that adipose wasting could precede muscle wasting in cancer cachexia through mechanisms dependent on adipose triglyceride lipase (ATGL) (72). C26 tumor-bearing mice exhibited a higher ability to induce adipose wasting, which could contribute to the progression of skeletal muscle wasting. Future work could investigate the differential adipose metabolism in C26 and CT26 tumor-bearing mice and how it influences skeletal muscle growth in the two models.

To illustrate potential mechanisms by which C26 and CT26 differentially impact skeletal muscle mass and function, we examined the intramuscular innate immune responses in different muscle types. We observed a higher level of muscle inflammation in C26 tumor-bearing mice compared to CT26 in both the SOL and EDL muscle. Immunohistochemistry on SOL showed that C26 inoculation induced the infiltration of CD68^+^ and CD206^+^ MΦ. Similarly, RT-qPCR also showed that C26 tumor-bearing mice had a significantly higher mRNA expression of the pan innate immune cell marker CD11b (*Itgam)* and the M2 macrophage marker CD206 (*Mrc1*) in the GAST. Previous studies have identified higher activity of tumor-associated macrophages (TAMs) in the tumor microenvironment, which promotes tumor proliferation and drives the production of cytokines such as IL-6 and CCL2 (73). It has also been shown that quadriceps from C26 tumor-bearing mice exhibited higher infiltration of both CXC chemokine receptor 1^+^ (CX3CR1^+^) MΦ and anti-inflammatory polarized M2-like MΦ (74). Although M2 MΦ are often considered to be anti-inflammatory/pro-regenerative and play an essential roles in the resolution of skeletal muscle inflammation, M2 MΦ secreted factors may also exert pro-tumorigenic roles and promote muscle wasting in the context of cancer (75, 76). Similarly, M2 MΦ have been shown to upregulate the TNF Receptor Associated Factor 6/NF-κB (TRAF6/p65) pathway in pancreatic cancer, which promotes the activation of Atrogin-1 and MuRF1 in skeletal muscle cells (77).

C26 tumor-bearing mice also exhibited a higher of intramuscular PMNs (Gr-1^+^ cells) in both SOL and EDL compared to CT26. Likewise, an elevation of both circulatory and intramuscular PMNs has been reported in C26 tumor-bearing mice, and such increase was not observed in CT26-bearing CD2F1 mice (34). In the same study, the authors also reported an increase in gene expression of transforming growth factor-β (TGF-β), hypoxia-inducible factor 1 subunit alpha (HIF1α), and IL-6 in SOL, which is limited to C26 tumor-bearing mice (34). Pathologically activated PMNs could potentially contribute to skeletal muscle wasting in cancer cachexia (78). Previously, research demonstrated that the polymorphonuclear myeloid-derived suppressor cells (PMN-MDSCs) promote skeletal muscle wasting by upregulating activin A signaling pathway in a mouse model of Lewis lung carcinoma (LLC) (79). In addition, we observed an increased expression of CHMP2A (*Chmp2a*) only in CT26 tumor-bearing mice. CHMP2A is required for autophagy and plays an important role in mediating autophagosome biogenesis (80, 81). Although the role of CHMP2A in mediating cancer cachexia remains unclear, excessive autophagy has been shown to promote muscle wasting (82). Moreover, autophagy could be involved in the infiltration and survival of PMNs in multiple cancer types (83). Collectively, the differential recruitment of innate immune cells to skeletal muscle could contribute to the different cachexic effects of C26 and CT26 tumors.

Despite clear evidence of cellular inflammation of the SOL and EDL of C26 tumor bearing mice we did not observe a transcriptional change of classical pro-inflammatory cytokines or chemokines such as IL-6 (*Il6*), MCP-1 (*Ccl2*), TNFα (*Tnf*), and IL1β (*Il1b*) in the GAST muscle. Intriguingly, previous research has shown a differential expression of cytokines and chemokines in the TA and GAST in C26 tumor-bearing mice. While the TA muscle had a significant increase in the expression level of MCP-1 (*Ccl2*), C-X-C motif ligand 1 (CXCL1, *Cxcl1*), and CXCL2 (*Cxcl2*), GAST only showed a massive increase in CXCL1 expression, revealing a differential inflammatory response to cancer progression in different muscle types (84). Although the upregulation of pro-inflammatory cytokines and chemokines are involved in the pathology of cancer cachexia, previous research have suggested that the progression of cachexia might not be dependent on the elevated expression of the cytokines (61, 78, 85). In addition, the change in the level of pro-inflammatory cytokines in muscle tissue could be time dependent.

Interestingly, we found that C26 and CT26 tumors also differentially mediated the expression of lipid biosynthesis enzymes in cachexic skeletal muscle. C26 tumors significantly increased the gene expression of 5-LOX (*Alox5*) while no such change was found in CT26 tumor-bearing mice. 5-LOX is the essential enzyme involved in the production of eicosanoids derived from the omega-6 arachidonic acid (ARA, 20:4n-6), including the leukotrienes (86). Overexpression of 5-LOX is observed in CRC tissues and contributes to cancer progression (87–89). In an acute model of myotoxin-induced skeletal muscle injury, it has been suggested that excessive 5-LOX activity may result in increased inflammation and impaired myofiber regeneration (90). Elevated 5-LOX activity has also been reported to drive myotube atrophy induced by glucocorticoids through promoting local production of the pro-inflammatory eicosanoid leukotriene B_4_ (LTB_4_) and activating the nuclear factor kappa B (NF‐κB) pathway (91). Additionally, it has been shown that the 4-weeks of treatment with the pharmacological 5-LOX inhibitor malotilate could alleviate skeletal muscle wasting in aging mice (91). On the other hand, we recently found that both malotilate and the 5-LOX activating protein (FLAP) inhibitor MK886 markedly interfered with the myogenic differentiation of C2C12 myoblasts (92). Therefore, the 5-LOX pathway may play a dual role in skeletal muscle plasticity under different circumstances. Nevertheless, our data suggests that chronically increased 5-LOX activity may contribute to skeletal muscle wasting in C26 tumor bearing mice

Unlike 5-LOX, we also observed a tumor-specific suppression of mRNA expression of leukocyte-type 12/15-LOX (*Alox15*) mRNA level in CT26 and C26 tumor-bearing mice, which is a key enzyme mediating SPM production and the resolution of inflammation (93). A reduced expression of 15-LOX-1, the human ortholog of murine 12/15-LOX, has been reported in human colorectal and pancreatic adenocarcinoma cell lines, which contributes to tumor survival and dysregulated tumor growth (94–96). However, studies on the influence of cancer cachexia on the expression of skeletal muscle 12/15-LOX have been limited. Here, we show that C26 tumors markedly diminished both the gene and protein level of 12/15-LOX in GAST. On the other hand, while CT26 tumors also reduced the mRNA expression of 12/15-LOX, no protein deficiency was found in CT26 muscles, indicating potential post-transcriptional mechanisms to compensate for 12/15-LOX function in CT26 tumor-bearing mice. It was recently reported that genetic deficiency of 12/15-LOX blocked the class switch of bioactive lipid mediators following acute muscle injury, resulting in impaired skeletal muscle regeneration following acute injury (92, 97). Transgenic mice lacking 12/15-LOX enzyme (*Alox15*^−/−^) also exhibited chronic basal skeletal muscle inflammation featured by increased infiltration of PMNs and MΦ (92). The diminished expression of 12/15-LOX in C26 tumor-bearing mice could contribute to the progression of muscle wasting through mediating inflammatory responses. Collectively, the distinct regulation of 5-LOX and 12/15-LOX expression in skeletal muscles could potentially contribute to the differential intramuscular immune responses and cachexic effects in CT26 and C26 tumor-bearing mice. Intriguingly, in LLC-induced cancer cachexia, despite a lower expression of 12/15-LOX protein in the GSAT muscle, the production of oxylipins derived from 12/15-LOX was apparently elevated, which has been suggested to contribute to progression of a cachexia phenotype by promoting oxidative stress (98). This could suggest a distinct pathophysiology of cancer cachexia induced by C26 and LLC. Further research should assess the intramuscular concentrations and biological roles of various bioactive lipid mediators derived from the 5-LOX and 12/15-LOX pathways in C26 and CT26 mice.

We also show that C26 and CT26 tumors differentially regulated signaling pathways involved in protein synthesis. Although no significant change was observed in the phosphorylation level of Akt at Ser473, C26 tumor-bearing mice tended to have a decreased phosphorylation level of p70S6K at Thr421/Ser424 while CT26 tumor-bearing mice did not. Akt/mTOR/p70S6K pathway plays a crucial role in regulating muscle protein synthesis, and repressed activity of Akt reduces muscle mass and contractile function (99, 100). Meanwhile, we also found that phosphorylation level of ERK1/2 was repressed in C26 tumor-bearing mice. ERK1/2 belongs to Mitogen-Activated Protein Kinase (MAPK) family and could positively regulate the activity of p70S6K in various tissues including cardiac muscles and retinal epithelium cells (101–103). While mTOR primarily targets Thr389 phosphorylation site of p70S6K, MAPK phosphorylates Thr421/Ser424 sites (104). It has also been shown that pharmacological inhibition of ERK1/2 represses p70S6K activity in C2C12 myotubes, resulting in a decreased myotube diameter (105). Consistently, we previously reported that phosphorylation of p70S6K induced by the eicosanoid prostaglandin F_2α_ (PGF_2α_) was mediated by an ERK dependent but Akt independent mechanism (106). The inhibited p70S6K phosphorylation at the Thr421/Ser424 sites in C26 GAST muscles could therefore be associated with the decreased activity of ERK1/2. Surprisingly, we observed an elevated phosphorylation of ribosomal protein S6, a well-established downstream effector of both the ERK and mTOR/p70S6K pathways, in C26 tumor-bearing mice at both Ser235/236 and Ser240/244 sites. Previous studies have shown that Akt/mTOR signaling could be largely impaired in colorectal cancer, indicated by the decreased phosphorylation level of p70S6K, S6, or 4E-BP1, which leads to decreased protein synthesis (23, 107, 108). However, a recent study showed that C26 tumor inoculation did not necessarily decrease the phosphorylation level of p70S6K or S6 despite significant loss of skeletal muscle mass (109). Moreover, increased phosphorylation of S6 does not directly reflect increased protein synthesis (110, 111). Our observation of the increased S6 activity could be associated with mTOR-related phosphorylation of p70S6K at Thr389 site in response to the release of amino acids following higher protein degradation (112–114). In addition, the increased activity of S6 could involve non-canonical pathways such as protein kinase A (PKA), which does not influence translational efficiency (115). Although it has been shown that PKA signaling was impaired in male mice after C26 inoculation, a differential regulation of PKA could occur due to mouse sex, age (2-month versus 10-month), and stage of cancer cachexia (Day 13 versus > Day 20) (116). Additionally, the activity of S6 phosphatase, primarily type 1 protein phosphatase (PP1), could be suppressed in colorectal cancer, which might also attenuate the suppression of S6 phosphorylation (117, 118). The potential regulatory network of ERK1/2, p70S6K, and S6 in the context of advanced CRC cachexia indicates a complex regulation of protein turnover pathways.

Notably, CT26 but not C26 tumor-bearing mice had a significantly upregulated phosphorylation of JNK at Thr183/Tyr185, which is another subfamily of MAPK (119). The JNK/c-Jun pathway could be associated with increased potential of metastasis and cancer stemness (120, 121). In the context of pancreatic cancer cachexia, it has been suggested that the activation of JNK signaling contributed to muscle wasting by activating the muscle-specific E3 ubiquitin ligases Atrogin-1 (*Fbxo32*) and MuRF1 (*Trim63*) (122). However, our model shows that despite the increased JNK phosphorylation in both CT26 and C26 mice, the mRNA expression of these muscle-specific E3 ubiquitin ligases was increased only in mice bearing C26 tumors. This result suggests other regulatory mechanisms of protein degradation pathway in CT26 tumor-bearing mice that counteracts the activation of JNK signaling. Furthermore, the greater mRNA expression of muscle-specific E3 ubiquitin ligases in C26 vs CT26 mice indicates a potentially higher protein degradation induced by C26 tumors via a JNK independent mechanism.

One limitation of this study is that circulating levels of pro-inflammatory cytokines were not measured. Although no change in cytokine expression was detected in GAST muscles, tumor-secreted cytokines could potentially be upregulated in plasma, contributing to systemic inflammation. We also did not examine the systemic or intramuscular profile of pro-inflammatory and anti-inflammatory/pro-resolving lipid mediators derived from various LOX-dependent pathways. Future studies should assess whether diminished 15-LOX and upregulated 5-LOX expression in skeletal muscle tissue of C26 tumor-bearing mice impact downstream concentrations of respective bioactive lipid mediators. Moreover, while recent studies indicate that male mice may be more prone to C26-induced cachexia, we used female BALB/c mice to compare the intrinsic difference of C26 and CT26 carcinoma (123, 124). Hence, future studies should investigate whether the differential effects of C26 and CT26 cells are impacted by sex. Further investigation could also assess the potential time-dependent changes in cytokine production in skeletal muscles. In addition, while we assessed the phosphorylation activity of major enzymes involved in protein synthesis and measured the expression of muscle-specific E3 ubiquitin ligases, the rate of protein synthesis or proteolysis was not directly measured. While our primary focus in the current study was the dynamics of skeletal muscle wasting, it could be worth examining the local cellular and molecular differences between C26 and CT26 tumors as well as how they differentially regulate adipose tissue wasting. Finally, future research could examine how muscle metabolism is mediated in C26 and CT26 tumor-bearing mice.

## CONCLUSION

In the current study, we found that both C26 and CT26 colon carcinoma cells induced skeletal muscle cell atrophy in a muscle-cancer co-culture model. However, in a mouse model of tumor inoculation, while CT26 tumors resulted in decreases in tumor-free body weight and muscle mass in GAST and TA, C26 inoculation caused a more significant loss of lean mass and a more severe impairment of skeletal muscle function. We show that C26 tumors led to a higher level of intramuscular inflammation than CT26, and C26 muscles had an increased expression of 5-LOX and a repressed expression of 15-LOX. Moreover, the two cell lines differentially regulate protein turnover pathways. Despite the upregulated activity of JNK signaling, CT26 exhibited a milder cachexia phenotype.

## Supplementary Material

Supplement 1

## Figures and Tables

**Table 1.** Primer sequences for RT-qPCR

Mouse Gene	Forward Primer 5′–3′	Reverse Primer 5′–3′
*Cd68*	ACTGGTGTAGCCTAGCTGGT	CCTTGGGCTATAAGCGGTCC
*Mrc-1*	GGCTGATTACGAGCAGTGGA	CATCACTCCAGGTGAACCCC
*Itgam*	TGGCCTATACAAGCTTGGCTTT	AAAGGCCGTTACTGAGGTGG
*Ccl2*	AGCTGTAGTTTTTGTCACCAAGC	GACCTTAGGGCAGATGCAGT
*Tnf*	ATGGCCTCCCTCTCATCAGT	TGGTTTGCTACGACGTGGG
*Il6*	TCCGGAGAGGAGACTTCACA	TTGCCATTGCACAACTCTTTTCT
*Il1b*	GCCACCTTTTGACAGTGATGAG	GACAGCCCAGGTCAAAGGTT
*Alox5*	ATTGCCATCCAGCTCAACCA	ACTGGAACGCACCCAGATTT
*Alox12*	GGATCCCTCAACCTAGTGCG	AGTCAAACTCCTCCTCCTTGC
*Alox15*	TGATGACTTGGCTGAGCGAG	TTCCCACCACGTACCGATTC
*Ptgs2*	AGCCAGGCAGCAAATCCTT	CAGTCCGGGTACAGTCACAC
*Fbxo32*	GGAAGTACGAAGGAGCGCCA	GATGGCAGTCGAGAAGTCCA
*Trim63*	CTGCAGAGTGACCAAGGAGAAT	GATGGCGTAGAGGGTGTCAA
*Chmp2a*	GTCGCTTAATCCGGAAACGG	GAACTAGCGGAGCCGAGAGA
*Ets*	CCAAGTGTTCATGCCACGTG	CGAGCGACTGCCACAAAAA
*Its-5.8S*	GCTCACACCCGAAATACCGA	TGCGTTCGAAGTGTCGATGA
*Vcp*	CTCCCTCCAAAGGCGTTCTT	TGGCCTCAGATTCCCCAAAC
*Gapdh*	CTTTGGCATTGTGGAAGGGC	CAGGGATGATGTTCTGGGCA
*Emc7*	AGAGCATGTCGGCTTCCTTA	ATCTTGCTCGCATTTTTCCTTTTG
