# Supplementary material for: C26 and CT26 colorectal cancer models exhibit divergent cachexia phenotypes, intramuscular inflammation, and protein turnover signaling
Source: bioRxiv. 2026 Apr 24:2026.04.21.719997. Preprint. [Version 1] doi: 10.64898/2026.04.21.719997 (PMC13131556; doi:10.64898/2026.04.21.719997)

bioRxiv preprint doi: https://doi.org/10.64898/2026.04.21.719997; this version posted April 24, 2026. The copyright holder for this preprint (which was not certified by peer review) is the author/funder, who has granted bioRxiv a license to display the preprint in perpetuity. It is made available under aCC-BY 4.0 International license.

Figure S1. The mRNA expression of pro-inflammatory cytokines and enzymes mediating ribosome biogenesis in skeletal muscle. RT-qPCR was used to determine the mRNA expression level of *Ccl2* **(A)**, *Tnf* **(B)**, *Il6* **(C)**, and *Il1b* **(D**) in GAST muscles. The rRNA fold change of ETS **(E)** and ITS-5.8S **(F)** was also measured in GAST muscles of healthy controls, CT26 tumor-bearing mice, and C26 tumor-bearing mice. Gene expression was normalized to the geometric mean of *Vcp*, *Emc7*, and *Gapdh*. *P<0.05 for difference compared to healthy controls, #P<0.05 for difference between C26 and CT26 tumor-bearing mice.

bioRxiv preprint doi: https://doi.org/10.64898/2026.04.21.719997; this version posted April 24, 2026. The copyright holder for this preprint (which was not certified by peer review) is the author/funder, who has granted bioRxiv a license to display the preprint in perpetuity. It is made available under aCC-BY 4.0 International license.

Supplemental Figure 1

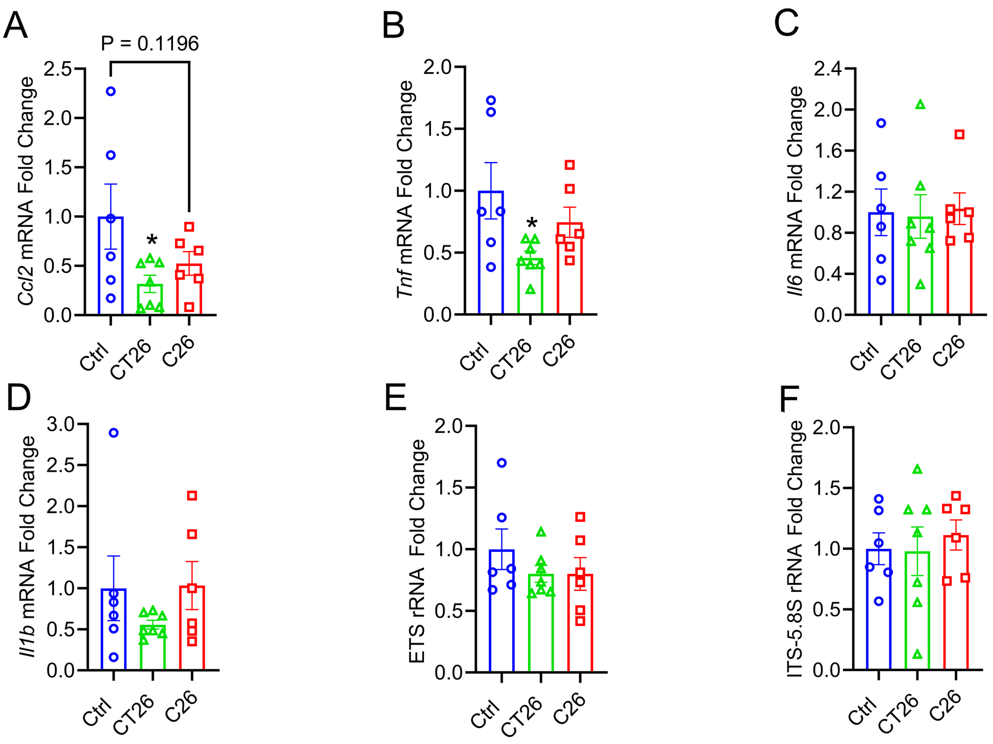

Supplement: Supplement 1 [file NIHPP2026.04.21.719997v1-supplement-1.pdf]
